# Micro-Raman Spectroscopy Analysis of Optically Trapped Erythrocytes in Jaundice

**DOI:** 10.3389/fphys.2020.00821

**Published:** 2020-07-10

**Authors:** Sanu Susan Jacob, Aseefhali Bankapur, Surekha Barkur, Mahendra Acharya, Santhosh Chidangil, Pragna Rao, Asha Kamath, R. Vani Lakshmi, Prathap M. Baby, Raghavendra K. Rao

**Affiliations:** ^1^Department of Physiology, Kasturba Medical College-Manipal, Manipal Academy of Higher Education, Manipal, India; ^2^Department of Atomic and Molecular Physics, Centre of Excellence for Biophotonics, Manipal Academy of Higher Education, Manipal, India; ^3^Department of Biochemistry, Kasturba Medical College-Manipal, Manipal Academy of Higher Education, Manipal, India; ^4^Department of Data Science, Prasanna School of Public Health, Manipal Academy of Higher Education, Manipal, India; ^5^Department of Physiology, Melaka Manipal Medical College, Manipal Academy of Higher Education, Manipal, India

**Keywords:** red blood cells, jaundice, Raman spectroscopy, bilirubin, hemoglobin

## Abstract

Derangements in bilirubin metabolism and/or dysfunctions in the hepato-biliary system lead to the unhealthy buildup of bilirubin in blood, resulting in jaundice. During the course of this disorder, circulating red cells are invariably subjected to toxic effects of serum bilirubin and an array of inflammatory compounds. This study aimed to investigate the vibrational spectroscopy of live red cells in jaundice using micro-Raman spectroscopy combined with optical-trap. Red cells from blood samples of healthy volunteers and patients with jaundice were optically immobilized and micro-Raman probed using a 785 nm diode laser. Raman signatures from red cells in jaundice exhibited significant variations from the normal and the spectral-markers were obtained from multivariate analytical methods. This research gives insightful views on how different pathologies can act as “stress-milieus” for red cells in circulation, possibly impeding their normal functions and also exasperating anemia. Raman spectroscopy, an emerging bio-analytical technique, is sensitive in detecting molecular-conformations *in situ*, at cellular-levels and in real-time. This study could pave way in understanding fundamental red cell behavior in different diseases by analyzing Raman markers.

## Introduction

Bilirubin is a yellow pigment produced in the body, primarily as a consequent-by-product of red blood cell (RBC) catabolism (Adamson and Longo, 2012). Senile RBCs at the end of their 120 days’ sojourn in circulation, are physiologically detected and hemolysed by the reticulo-endothelial system. This process occurs chiefly in the spleen, causing the release of hemoglobin (Hb), the principle RBC component (Korolnek and Hamza, 2015). When the tetra-pyrrolic moiety of hemoglobin, called heme, breaks down, it generates plasma-insoluble unconjugated bilirubin (UCB) (Schiff et al., 2011). To render it plasma-soluble, UCB is reversibly-bound with plasma albumin and is transported to the liver to undergo a process called conjugation. In the liver, UCB is conjugated with glucuronic acid (Levitt and Levitt, 2014). Plasma-soluble conjugated bilirubin (CB) is then released through the bile into the intestine, from where it is excreted and eliminated from the body through the bowels and bladder in the form of stercobilinogen (Erlinger et al., 2014).

In any circumstances of increased hemolysis, hepatic dysfunction or even biliary obstruction, there ensues a build-up of bilirubin in blood, resulting in a condition termed hyperbilirubinemia, which is clinical jaundice (Fargo et al., 2017). Normal serum bilirubin levels are not known to pose any implications for general health, rather, is found to be valuable for possessing antioxidant properties in the vasculature (Park, 2016). However, uncontrolled and/or untreated hyperbilirubinemia is toxic and can cause serious deleterious effects that require immediate and appropriate life-saving treatments (Hansen, 2001). Currently, there exist numerous reports that describe the lethal effects of jaundice, but most studies are directed toward its effects on the nervous system (Shapiro, 2003; Shi et al., 2019). There exist fewer reports of bilirubin toxicity on blood components. It has been recognized that bilirubin liberated from RBC destruction, if present in larger than physiological quantities in circulation, can trigger toxic effects on other circulating RBCs. This can result in augmenting the production of more bilirubin, the whole course transitioning into a potentially vicious cycle (Lang et al., 2015). In addition to bilirubin, levels of a multitude of inflammatory markers and acute phase reactants escalate in the serum, such as endotoxins, tumor necrosis factor-α, IL-6, and C-reactive protein (Padillo et al., 2001). These systemic inflammatory cytokines are themselves known to reduce the life-span of RBCs, through a programmed process called eryptosis (Pretorius, 2018).

The focus of this study was to investigate the chemometrics of the Raman spectra of RBCs from blood samples of patients with jaundice, keeping the cells as alive as possible. This required the utility of a tool that demands only the bare minimal sample-preparation procedures, reagents or fixatives, which could themselves be potential sources of cell-injury or cell-modification (Sato et al., 2018). Keeping these viewpoints into consideration, we made use of a home-built micro-Raman spectroscopy system coupled with laser-tweezers to investigate the biochemical characteristics of RBCs (Bankapur et al., 2010). Raman spectroscopy has been gaining popularity in its application in biomedicine as this single-cell technique is tremendously sensitive to very subtle molecular changes within the cell, records vibrations of molecular bonds in its original state instantaneously and does not employ the usage of any chemical markers (Kumamoto et al., 2018). This research aimed to comprehend the molecular changes of RBCs, in a “jaundice” environment.

## Materials and Methods

### Ethics Statement

This study was approved (IEC 02/2002) by the Institutional Ethics Committee of Kasturba Medical College, Manipal Academy of Higher Education, Manipal and all experimental procedures conformed with the Ethical Committee guidelines. Written informed consent was obtained from all the volunteers. Blood samples of confirmed hyperbilirubinemic patients were collected from the laboratory of Clinical Biochemistry. The requirement for informed consent was waived concerning the acquisition of patients’ samples.

### Blood Sample Collection and RBC Preparation

Four milliliter anticoagulated blood samples were collected in EDTA-containing BD Vacutainers from healthy volunteers (*n* = 10). Blood samples of hyperbilirubinemic patients (*n* = 28) in EDTA were collected from the laboratory of Clinical Biochemistry. Whole anticoagulated blood sample was centrifuged (Labinet Spectrafuge 7M) at 5000 rpm for 5 minutes. Following centrifugation, the plasma and buffy-coat layers were aspirated off using a micropipette. One microliter hematocrit (Hct) was pipetted out and suspended in 2 milliliter phosphate-buffered saline (PBS)-filled Eppendorf tube to obtain a suspension of live RBCs. PBS was procured from Sigma Aldrich, India.

One microliter of RBC suspension in PBS was pipetted out from the Eppendorf tube onto the custom-made sample chamber. A total of 379 raw spectra were recorded from 379 individual RBCs, of which 147 spectra were recorded from the blood samples of healthy volunteers and 232 from that of patients with jaundice.

### Experimental Set-Up

**FIGURE 1:**
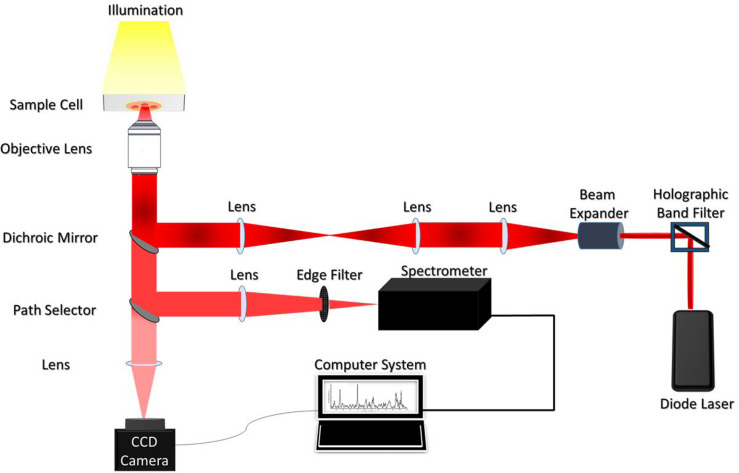
Schematic diagram of the Raman Tweezers set up.

The illustration of the single-beam micro-Raman spectrometer coupled with optical tweezers has been shown in Figure 1. The system comprised of an inverted microscope (Nikon Eclipse Ti-U, Japan) coupled with a 785 nm wavelength-emitting diode laser (Starbright Diode Laser, Denmark) and a spectrograph (Horiba Jobin Yvon iHR320). The expanded laser-beam overfills the back aperture of a 100X, 1.3NA microscope objective (Nikon, Plan Fluor) to create a sharp focal-spot at the sample plane. Single RBCs suspended in PBS were optically-trapped under this focal-spot and simultaneously Raman-excited. The scattered light from the individual RBCs was collected from the very same objective and fed into the spectrograph using f-matching optics. The spectrograph was equipped with a 1200 groves/mm grating and a liquid nitrogen-cooled charged couple device (Symphony CCD-1024x256-OPEN-1LS) detector, with 1024 × 256 pixels. An optical edge-filter (Razor edge LP02-785RU-25, Semrock, United States) was placed just in front of the spectrograph for the removal of Rayleigh scattered radiations. The comprehensive description of the experimental set-up has been published in earlier studies (Bankapur et al., 2010, 2014).

### Data Acquisition

Utilizing the above setup, RBCs were optically immobilized and probed using a 785 nm diode laser with an excitation-power of 10mW. RBCs were positioned at the focus of the laser beam by adjusting the manual stage. Five accumulations were recorded per RBC with the exposure/acquisition time of 60 seconds. Raman spectra of RBCs were procured within the spectral range of ∼600 to 1,800 cm^–1^. This spin-sensitive-region is observed to deliver maximum information on the “oxygenation-state” of Hb (De Luca et al., 2008).

### Data Pre-processing

Origin (OriginLab Corp., Northampton, MA, United States), MATLAB (MATLAB^®^ 7.0) and GRAMS (Grams/AI, PLS Plus IQ) software were employed for the pre-processing of the accrued RBC spectra. 379 raw spectra that included those from the healthy, as well as the jaundice groups, were pre-processed.

The pre-processing steps involved the exclusion of spurious noises/cosmic-spikes, spectral smoothening, baseline-correction, and vector-normalization. The data-points that corresponded to cosmic-spikes were deleted using “Origin.” Using GRAMS, the raw spectra were smoothened out by second-order polynomial Savitzky-Golay moving-average-technique. Spectral normalization was accomplished by 2-norm standard-vector normalization (Barkur et al., 2015) using GRAMS. Multiple spectral baseline-correction was effected by employing asymmetric least-squares (AsLS) fitting approach proposed by Eilers (2003).

### Statistical Analysis

Multivariate statistical analyses were carried out using GRAMS-AI and CRAN R 3.6.1 (©2019 The R Foundation for Statistical Computing) packages. The intensity of bands in 600–1800 cm^–1^ range of the Raman spectra was analyzed.

The goal was to choose seamless statistical tools for the classification of acquired data, based on variations in spectral features. In this study, for effective categorization, we had performed partial least squares based discriminant analysis (PLS-DA), a supervised diagnostic model, to sharpen variations between the generated principal components (PCs) using Grams IQ spectroscopy software. The PCs were rotated such that a maximum separation among classes was obtained. Additionally, PLS-DA helped to identify the Raman signatures responsible for classification. This was followed by a non-parametric variant of Multivariate ANalysis Of VAriance (MANOVA) to re-affirm the statistically significant difference in the mean value of the measurements captured at different wavelengths across the healthy and jaundice samples. Subsequently, a paired Wilcoxon *post-hoc* test with Bonferonni correction was used to facilitate multiple comparisons. The statistical significance was defined as a *p* ≤ 0.05.

## Results

### Raman Spectra of RBCs From Healthy and Jaundice Blood Samples

**FIGURE 2:**
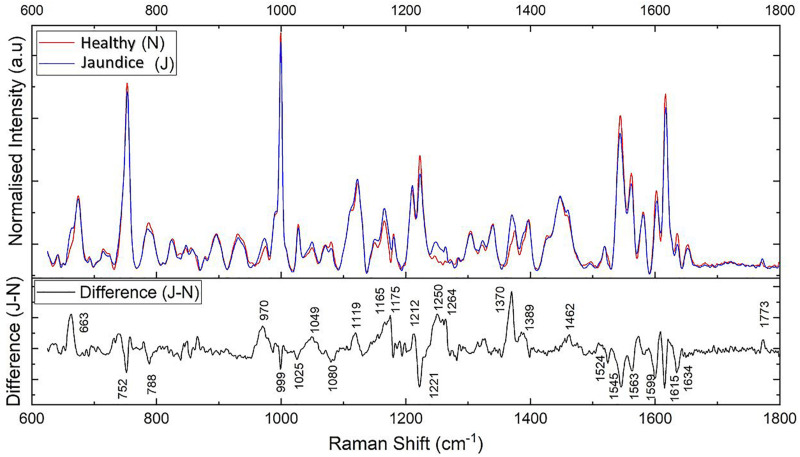
Illustrates the averaged Raman spectra of RBCs from healthy (N) and jaundice (J) samples in the spectral region 600–1,800 cm^–1^ **(on top)** and the difference (J–N) between the averaged spectra between the healthy and jaundice blood samples **(below)**.

The pre-processed spectra were first overlaid together separately for each group (Supplementary Figure S1). This exhibited the overall trend of the Raman peaks for the healthy and jaundice groups separately. Figure 2 gives the overlaid averaged Raman spectra of both the healthy and jaundice groups. The spectral bands were assigned as was described in earlier studies. Since RBCs comprised predominantly of Hb protein, spectral data were dominated primarily by the Raman signatures from heme. There also appeared to be contributions from other organic components as well, such as aromatic amino acids, amide bonds and –CH/-CH_2_ side-chains of globular proteins.

**FIGURE 3:**
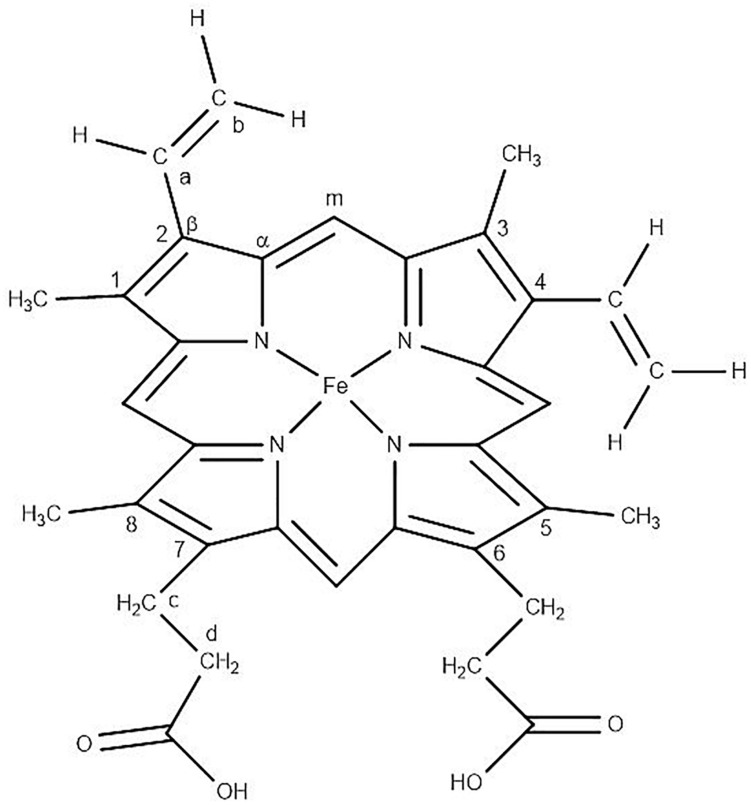
Chemical structure of “heme” in RBC displaying the Fe-protoporphyrin-IX with four pyrrole rings, four methyl (CH_3_) groups, two propionate side chains and two vinyl (−CH = CH_2)_ groups.

The averaged spectra exhibited multiple differences in terms of both intensity changes as well as frequency shifts. RBCs from the jaundice group displayed increase in intensity of Raman frequencies at 975, 1,049, 1,123, 1,165, 1,175, 1,212, 1,248, 1,258, 1,264, 1,370, 1,375, 1,389, 1,460, and 1,771 cm^–1^ and a decrease in intensity at wavelengths 752, 788, 999, 1,025, 1,080, 1,223, 1524, 1,545, 1,563, 1,602, 1,617, and 1,636 cm^–1^ when compared to those from RBCs of the healthy group. Additionally, the averaged Raman spectrum in the disease group exhibited a new peak at 663 cm^–1^ and a 2–3 cm^–1^ frequency shift in 975 and 1,248 cm^–1^ bands. The detailed vibrational assignments of the same have been summarized in Table 1 and Figure 3 illustrates the detailed chemical structure of heme.

**TABLE 1:** Assignments of the significantly different Raman peaks of RBCs of healthy and jaundice samples.

Healthy (cm^–1^)	Jaundice (cm^–1^)	Changes	Assignments	*P*-value
–	663	*New Peak*	p:C-S str (gauche)	<0.00001
752	752	↓	pyrrole ring def (out-of-plane), Tryptophan	<0.00001
788	788	↓	pyrrole ring breathing	<0.00001
975	973	↑ and Shift	*ν*(C_*c*_–C_*d*_)	<0.00001
999	999	↓	Phenylalanine	<0.00001
1,025	1,025	↓	Phenylalanine	<0.00001
1,049	1,049	↑	ν(O = O), δ(= C_*b*_H_2_)_*asym*_	<0.00001
1,080	1,080	↓	p: C-N str	<0.00001
1,122	1,123	↑	*ν*(C_β_ –C_1_)_*sym*_	<0.00001
1,165	1,165	↑	Pyrrole half-ring vibration	<0.00001
1,175	1,175	↑	Pyrrole half-ring vibration	<0.00001
1,212	1,212	↑	Methine C_*m*_–H def	<0.00001
1,223	1,223	↓	Methine C_*m*_–H def	0.003
1,246	1,248	↑ & Shift	p:Amide III (disordered)	<0.00001
1,258	1,258	↑	p:Amide III (disordered)	<0.00001
1,264	1,264	↑	p:Amide III (disordered)	<0.00001
1,370	1,368, 1,375	↑	Pyrrole half vibration	<0.00001
1,389	1,389	↑	Pyrrole half vibration	<0.00001
1,460	1,460	↑	δ(= C_*b*_H_2_)_*sym*_, p:δ(CH_2_)	<0.00001
1,524	1,524	↓	ν(C_β_ −C_β_)	<0.00001
1,545	1,545	↓	ν(C_β_ −C_β_)	<0.00001
1,563	1,563	↓	ν(C_β_ −C_β_)	<0.00001
1,602	1,602	↓	*ν*(C_α_ −C_*m*_)_*asym*,_ν (C_*a*_ = C_*b*_)_*venyl*_	<0.00001
1,617	1,617	↓	*ν*(C_α_ −C_*m*_)_*asym*,_*ν*(C_*a*_ = C_*b*_)_*venyl*_	<0.00001
1,636	1,636	↓	*ν*(C_α_ −C_*m*_)_*asym*_	<0.00001
1,771	1,771	↑	p: C = O str	0.07

*p, protein; sy, symmetric; asym, antisymmetric; str, stretch; ν and δ, in-plane modes; str, stretching, def, deformation. RBCs from healthy volunteers and jaundice patients suspended in PBS were optically-trapped and excited with a 785 nm diode laser and their Raman spectra were captured. The given table lists the frequencies of Raman signals that had displayed increased, decreased and shifted peaks. The assignments of the Raman peaks were carried out based on previous studies.*

### Multivariate PLS-DA and Factor Analysis Approach

**FIGURE 4:**
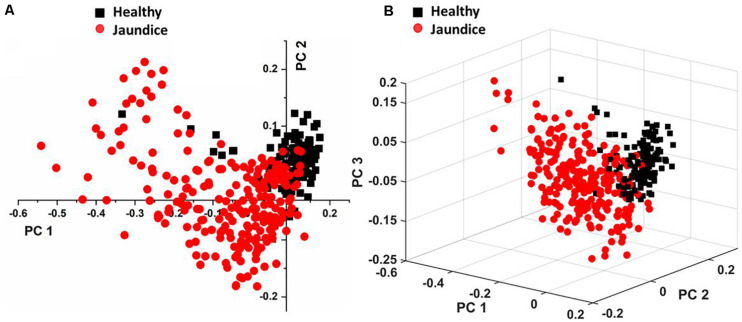
Illustrates PLS-DA scores plots of **(A)** first two PCs and **(B)** first three PCs.

Figure 4A displays the two-dimensional plot of the first two PCs: PC_1_ and PC_2_, and Figure 4B gives the 3-dimensional plot of the first three PCs: PC_1_, PC_2_, and PC_3_. Both scores-plots (generated by Grams IQ) exhibited a tight clustering of RBCs from the healthy group, with negligible variance. However, RBCs from the jaundice samples were scattered farther away from those of the healthy group to form a different and wider-distributed group. The variations in the PCs appeared significantly different for classification as healthy and “stressed” due to jaundice. The variance captured in this approach, taking into account all the wavenumbers in the spectra between the groups, was 31.08%.

**FIGURE 5:**
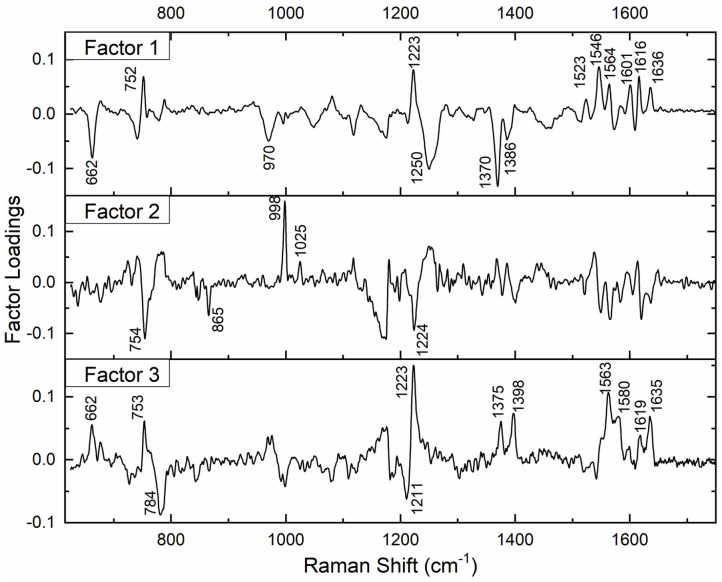
Spectral loadings of PC factors (Factor 1, Factor 2, and Factor 3) between RBCs of healthy and jaundice samples.

Subsequently, we performed factor load analysis, taking into consideration the first three factors (Figure 5). It was observed that all the Raman peaks that showed positive peaks in Factor-1 loading allied to their corresponding peaks of increased intensities in Figure 2. Similarly, all the Raman signals that showed negative peaks in Factor-1 loading allied with their corresponding peaks with decreased intensities in Figure 2.

Factors 2 and 3 picked up the next level variations in the spectra, chiefly of Raman peaks at 752, 999, 1,025, and 1,223 cm^–1^.

### RBC Raman Markers Detected in Jaundice

Averaged spectra, PLS-DA and the factor analysis extracted the Raman frequencies that exhibited substantial variations between RBCs from healthy and jaundice blood samples. The observed variations and their assignments have been described below under the four specific band regions, viz., core size/spin-state marker region, pyrrole-ring stretching region, methine C-H deformation region and the low wavenumber region (Wood et al., 2001). It has to be noted that the Raman peaks were assigned based on already existing data from previously conducted studies.

#### Core Size or Spin State Marker Band: 1,500–1,650 cm^–1^

This specific band region comprises Raman signatures that were scattered from the C-C bonds in the porphyrin ring and rely on the spin-state of the iron atom (Wood and McNaughton, 2002). It is an important spectral range to explore, as it describes the “oxygenation” (oxy) state of Hb (Lukose et al., 2019). The Raman peaks at 1,524, 1,545, 1,563, 1,602, 1,617, and 1,636 cm^–1^ exhibited a decrease in their intensities in the jaundice samples when compared to their corresponding peaks from the healthy samples. This was an indication that Hb in jaundice was largely present in their “deoxygenated” (deoxy) state, with less bound oxygen (Rao et al., 2009). When iron conforms from the low-spin oxy-state to the deoxy-state, the iron is displaced a few nanometers out of the porphyrin plane as a result of electron withdrawal (Wood et al., 2001; Burchett et al., 2017). This “switch” becomes reflected in the vibrational modes of the porphyrin rings and marks the changes in the above-mentioned peaks. There appeared to be a significant reduction in the intensity of the peak at 1,636 cm^–1^, a phenomenon attributed to porphyrin-doming (Rao et al., 2009). This peak is considered as the Raman-marker characterizing oxygen-concentration. An intensity decrease of this peak indicates a decline in the oxy-configuration of heme (Luo et al., 2015).

#### Pyrrole-Ring Stretching Band: 1,300–1,400 cm^–1^

This Raman band region signifies the “oxidation state” of iron in the pyrrole ring and comprises heme-aggregation bands (Ong et al., 1999). In jaundice, there appeared to be a prominent increase in the intensity of the heme-aggregation Raman peak at 1,370 cm^–1^ (Wood et al., 2005) accompanied by enhanced peaks at 1,375 and 1,389 cm^–1^. The latter peaks are designated to pyrrole deformation/breathing modes (Dybas et al., 2018). This indicated that RBCs in the “jaundice environment” produced heme-aggregates within themselves. There appeared to be a shift in the 1,370 cm^–1^ (oxy state) peak to 1,375 cm^–1^ (deoxy state) in jaundice, ascertaining lesser oxy states in jaundice (Lukose et al., 2019).

#### Methine C-H Deformation Band: 1,200–1,300 cm^–1^

This is the spectral zone assigned to methine C-H bonds in heme molecules. These bonds are sensitive to minute changes in Hb conformations and are governed by porphyrin-iron associations. The high sensitivity of these bonds has been described to be because of their juxtaposition with the protein subunits (Wood et al., 2001). Thus, Hb conformational-variations alter the deformation angle of C-H vibrations instantaneously (Wood et al., 2007). In this study, the wavenumbers that displayed enhanced intensity in jaundice were the peaks at 1,212, 1,248, and 1,264 cm^–1^ which were also accompanied by a fall in intensity at the peak 1,223 cm^–1^, consequent to changes in the methine C-H deformation regions. We also report a shift in the wavenumber 1,246 cm^–1^ to heme aggregation marker 1,248 cm^–1^ in jaundice (Lemler et al., 2014).

#### Low Wavenumber Band: 600–1,200 cm^–1^

This spectral region encompasses the bands for pyrrole-breathing, pyrrole-deformation modes and RBC membrane stability. In jaundice, there was a shift of the Raman peak at 973 to 975 cm^–1^ with a significant increase in its intensity along with a shouldering of 675 cm^–1^ pyrrole-deformation band with a new peak at 663 cm^–1^. These occurrences indicate heme-aggregation, as a consequence of protein denaturation, under “stressful” environments (Wood et al., 2005; Lukose et al., 2019). The 752 cm^–1^ porphyrin breathing mode peak, a crucial spectral-marker that confers the integrity of Hb (Deng et al., 2005), shows reduced intensity in the jaundice group. The peak 788 cm^–1^, a deoxy Raman marker, has taken a noticeable decrease in its intensity in jaundice samples (Barkur et al., 2017). The 999 and 1,025 cm^–1^ Raman peaks, originating from phenylalanine, exhibited a reduction in their strengths, signifying the breakdown of both Hb (Wu et al., 2011; Raj et al., 2013) as well as cell membranes (Lippert et al., 1975; Goheen et al., 1993). Peak 1,122 cm^–1^, emanating from the RBC membrane, is caused by the trans conformation vibrations of the C-C frame, the height of which has intensified and so does the peak at 1,080 cm^–1^, signifying diminished membrane deformability (Lukose et al., 2019). Changes in intensity at 1,165 and 1,175 cm^–1^ attributes to deformation in pyrrole ring of Hb (Gautam et al., 2018).

### PCA of the 26 Detected Wavenumber-Bands

In total, there were 26 Raman bands (Table 1) detected by the spectral analysis approach that displayed a substantial difference between the healthy and jaundice samples. Accordingly, PCA was conducted again, taking into consideration the intensity-scores of just these 26 Raman peaks. The analysis captured a variance of 60.54% for the first three PCs, much higher than that obtained (31.08%) when the whole region of the spectra was considered.

### Non-parametric MANOVA Approach to Raman Spectral Markers

We then proceeded to explore whether there existed a significant difference in the mean intensity-scores across the two groups for the 26 Raman bands identified in the spectral analysis by a MANOVA-based approach using CRAN 3.6.1. The packages utilized for this analysis were heplots (Fox et al., 2018), npmv (Wood et al., 2007), and MVN (Korkmaz et al., 2014). The advantage of implementing this method was that it made use of dependencies between the wavelengths and enabled joint acceptance or rejection of the hypothesis. When we tested the assumptions of MANOVA, it was observed that the assumptions of multivariate-normality as well as the equality of covariance-matrices that were associated with the two groups were violated. Hence, we made use of a non-parametric variant of MANOVA. Since the data represented intensity-scores across different Raman peaks corresponding to two populations, the McKeon approx. for Lawley Hotelling Test (*p* < 0.00001) was considered. The results indicated a significant difference between the average values of the intensity-scores across the healthy and jaundice samples. As a *post-hoc* analysis, paired Wilcoxon test with Bonferonni corrections was implemented to identify the wavelengths that accounted for significant differences in the intensity-scores across the two populations. The p-values that were derived are presented in Table 1.

Excluding Raman peak at 1,223 cm^–1^ (*p* = 0.003), that emanated from the methine C-H deformation band and 1,771 cm^–1^ (*p* = 0.07) from the *ν*(C = O) peak of proteins, the intensity scores of all the other Raman bands detected by spectral analysis exhibited a highly significant difference (*p* < 0.00001) between the healthy and jaundice samples.

Therefore, the “vitality” or “normality” of RBCs can be deduced by just analyzing these specific Raman peaks, rather than assessing all the RBC Raman bands across the spectrum. The variations in these peaks convey information regarding deformations/distortions in both cytoplasmic Hb as well as the RBC membrane. This study consequently informs us that under pathologies relating to jaundice, RBCs in circulation, although looking “morphologically” healthy, may not always be “chemometrically” healthy.

## Discussion

Jaundice (derived from the French word “jaune” which means yellow) is the yellowish discoloration of the sclera, skin and mucous membrane, caused by the build-up of serum bilirubin, above 2 mg/dL, and nearly always signifies the existence of an underlying infection related to the derangement of bilirubin metabolism. Its persistence is associated with a substantial increase in morbidity and mortality (Anand and Garg, 2015). Our body produces close to 4 mg/kg bilirubin every day (Schiff et al., 2011). Normally, the total serum bilirubin level never exceeds 1 mg/dL, of which UCB is less than 0.8 mg/dL and CB levels is less than 0.3 mg/dL (Zieve et al., 1951; Werner et al., 1970). There exists a myriad of clinical pathologies that results in jaundice. Unconjugated hyperbilirubinemia results primarily due to excessive hemolysis, while hyperbilirubinemia caused by excessive CB, develops in hepatocellular diseases and obstructions of the biliary system. In the latter cases, there can be a rise in both forms of bilirubin (Fargo et al., 2017). In this study, we have included patients diagnosed with hyperbilirubinemia, irrespective of its cause.

Hyperbilirubinemia can be toxic to circulating RBCs, causing morphological and metabolic impairments, eventually leading to further hemolysis, aggravating hyperbilirubinemia and intensifying anemia (Lang et al., 2015). Bilirubin toxicity on RBCs has been found to occur in a concentration and temperature-dependent manner (Brito et al., 2000). During the course of the disease, jaundice is accompanied by elevated levels of several enzymes and inflammatory-cytokines in serum (Padillo et al., 2001). This adds up to the already existing distress on healthy RBCs (Pretorius, 2018). Taking these facets into account, we attempted to examine the biochemical status of live RBCs in jaundice employing Raman spectroscopy. Since this analytical technique is both reagent and fixation-free, it rules out any concerns regarding possible cell modifications or damage caused by the analytical processes itself (Sato et al., 2018). Because RBCs are delicate and extremely sensitive cells, it was very important to ensure utmost care in the management of samples during the procedures that might themselves lead to chemometric variations, hemolysis or even eryptosis. We had also ensured to conduct the experiments immediately on sample-procurement.

Our results showed distinct variations in the biochemical signatures of RBCs in jaundice, when compared to the normal. Since RBCs are simple organelle-less cells with a highly deformable cell membrane that holds abundant cytoplasmic Hb, the obtained Raman spectral features were derived from these two sources. The overlaid averaged Raman spectra from the groups displayed Raman peaks that had their intensities altered and a few peaks that had undergone a shift (Figure 2 and Table 1). It is to be noted that the Raman peaks were assigned to specific biochemical components of the RBC in accordance with earlier studies and in this study we report how these Raman signatures altered in clinical jaundice. Spectral analysis and the various multivariate analytical methods employed in this study indicate chemometric modifications of RBCs in jaundice, when compared to RBCs of the healthy group. The variations (section “RBC Raman Markers Detected in Jaundice”) were noted in Raman peaks that represented the “oxygenation status” of Hb. The heme in jaundice exhibited largely a “deoxy” status when compared to the more “oxy” states in the healthy population. This was accompanied by a decline in the frequencies signifying “oxygen saturation” in the jaundice samples. Heme-aggregation-Raman-markers were manifested in jaundice along with a fall in peaks that depicted Hb stability. The spectra also exhibited alterations in Raman peaks portraying RBC membrane stability. The non-parametric MANOVA method served as an aid in the identification of the Raman signatures that could distinctly (*p* < 0.00001) capture the significant differences in the intensity scores across healthy and jaundice populations (Table 1). PCA of these identified Raman signatures captured better variance between the groups.

These results exhibit enough evidence to establish that in jaundice, intact and healthy-appearing RBCs in circulation, can experience biochemical alterations similar to those seen when under distress. The probable reasons could be the hostile milieu created by the disease itself such as hyperbilirubinemia, presence of pathogens and/or products related to the immune response that alters the physiological state of blood, thereby experiencing a shortened life-span. The intention of this study was to report the Raman spectroscopy features of RBCs in jaundice and analyze the generated findings, irrespective of the disease or it’s cause. An attempt was made to correlate Raman spectral changes with serum bilirubin levels in jaundice. But the results with this group yielded less correlation (Supplementary Figure S2 and Supplementary Table S1). Further exploratory studies are required to investigate the correlation of bilirubin levels and other systemic factors with RBC Raman spectral variations in specific pathologies.

## Conclusion

It is challenging to unearth the exact biochemical status of RBCs, as existent *in-vivo*, considering their extremely delicate and sensitive nature to even minuscule changes in its environment. Therefore, on a routine basis, RBC pathology is clinically assessed by a collective assemblage of tests that encompasses the estimation of its counts, Hb and bilirubin levels and analysis using peripheral smears. Unlike routine assays or even other high-end analytical techniques such as flow cytometry, Raman spectroscopy does not demand labels and is unique by being more sensitive and specific. The Raman-scattered-light from a particular substance characterizes its own exclusive “chemical-fingerprint.” In recent times this tool has been increasingly employed in hematological research for elucidating structural information from hemes, demonstrating the existence of characteristic molecular signatures from them under different conditions of stress such as oxidation and energy depletion, disorders such as thalassemia and diabetes, and diseases such as malaria and carcinomas. This study is merely a commencement to understand the chemometric behavior of RBCs in jaundice and needs to be extrapolated further to comprehend its characteristics in specific diseases/conditions that lead to it.

## Data Availability Statement

The raw data supporting the conclusions of this article will be made available by the authors, without undue reservation, to any qualified researcher.

## Ethics Statement

The studies involving human participants were reviewed and approved by the Institutional Ethics Committee, Kasturba Medical College and Kasturba Hospital Registration no. ECR/146/Inst/KA/2013/RR-16. The participants provided their written informed consent to participate in this study.

## Author Contributions

SJ, AB, PR, and SC conceived and designed the study. SJ and AB performed the experiments, interpreted the results, and wrote the manuscript. SB and MA contributed to performing the experiments. RR and PB contributed to the conception and design of the project and the interpretation of results. AK and RL contributed to the statistical analysis, interpretation of results, and wrote the manuscript. All authors contributed to the article and approved the submitted version.

## Conflict of Interest

The authors declare that the research was conducted in the absence of any commercial or financial relationships that could be construed as a potential conflict of interest.
