# Supplementary material for: Micro-Raman Spectroscopy Analysis of Optically Trapped Erythrocytes in Jaundice
Source: Front Physiol. 2020 Jul 10;11:821. doi: 10.3389/fphys.2020.00821 (PMC7366392; doi:10.3389/fphys.2020.00821)
Supplement: Supplementary file 1 [file Data_Sheet_1.PDF]

Supplementary Material

1 Supplementary Results

We present in this supplementary section the figures displaying the Raman spectra recorded from the healthy and jaundice groups (Supplementary Figure 1) overlaid separately, PLS-DA score plot of PC1 and PC2 values of all the jaundice patients (Supplementary Figure 2) and a table (Supplementary table 1) depicting the relevant anthropometric and haematological data of the jaundice patients.

2 Supplementary Figures and Tables

2.1 Supplementary Figures

2.11 Supplementary Figure S1: After the pre-processing of all the Raman spectra from each group (healthy and jaundice), they were overlaid for each group. This figure depicts the cell-to-cell information from RBCs of the healthy and jaundice samples separately. Figure 2 of the main manuscript is derived by averaging the following spectra from each group and overlaying the average spectra of both groups.

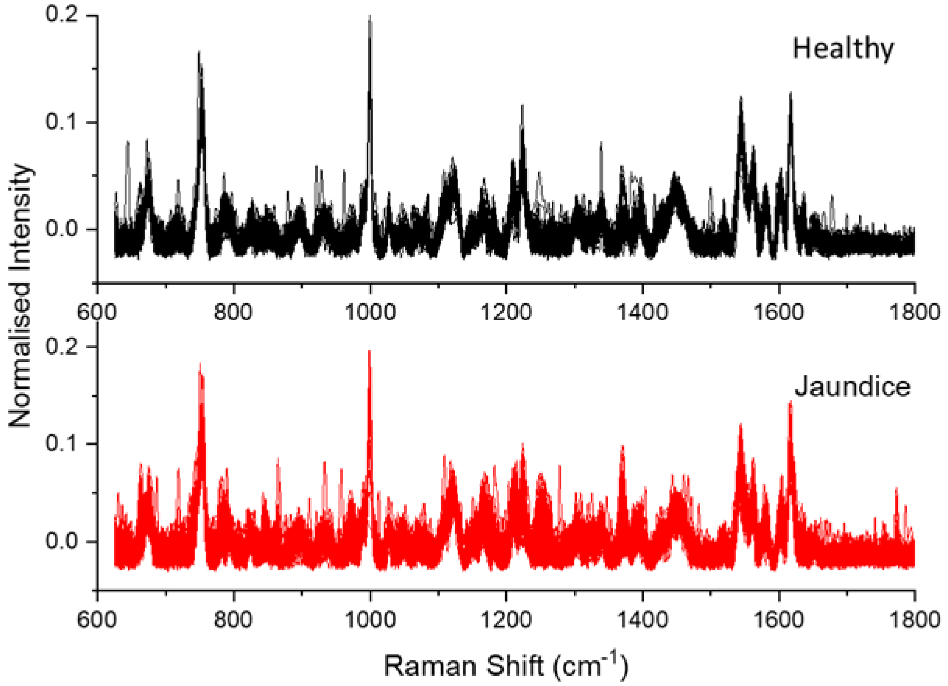

Supplementary Figure S1: Cell-wise RBC Raman spectra of healthy **(n=10)** and jaundice **(n=28)** blood samples. The RBCs were optically immobilized as well as probed using a 785nm diode laser with an excitation-power of 10mW. 5 accumulations were recorded per RBC and the

exposure/acquisition time was of 60 seconds duration. The Raman spectra were procured within the spectral range of ~ 600 to 1,800 cm^{-1}.

2.12 Supplementary Figure S2: We attempted in examining for any correlations between the Raman spectroscopy details of RBCs in the jaundice samples with their bilirubin values. This figure illustrates the patient-wise PLS-DA plot of the PC1 and PC2 scores from the RBCs in the jaundice group. The hematological parameters of each patient is displayed in supplementary table S1. Further studies are necessary to effectively study the correlation between the extent of jaundice and the RBC Raman characteristics.

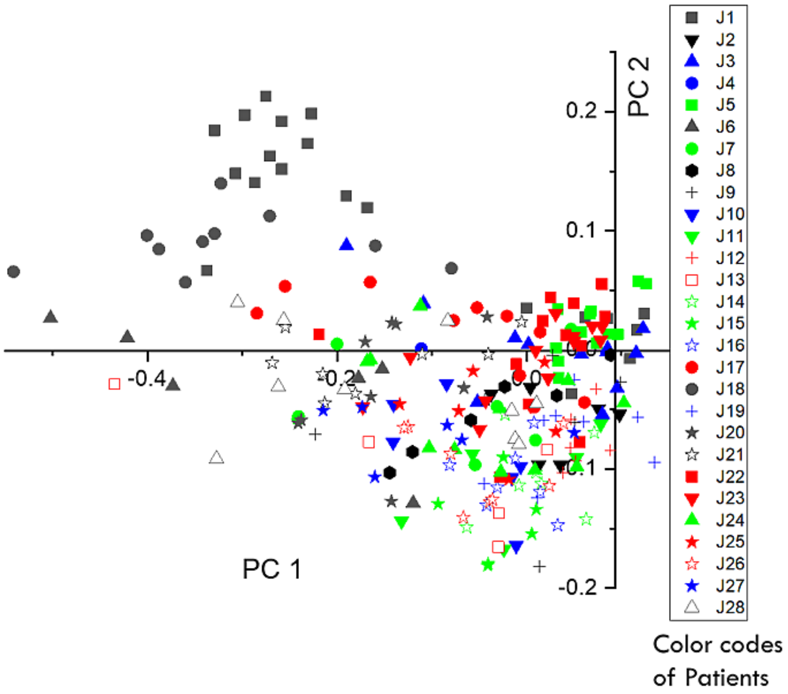

Supplementary Figure S2: Patient-wise PLS-DA plot of PC1 and PC2 scores. The scores of each patient (whose hematological parameters are given in supplementary table 1) is color-coded. The plot does not express a clear correlation between the Raman spectroscopy features of RBCs and the serum bilirubin levels of the jaundice samples. The scores of all samples seem to form a cluster, irrespective of their serum bilirubin levels. Very few scores had formed a wide scatter away from the cluster.

2.2 Supplementary Table

Supplemental Table S1

Blood samples of healthy volunteers **(n=10)** were collected in EDTA-anti-coagulated BD vacutainers. Since the volunteers did not show symptoms of anemia or clinical jaundice, their hematological parameters were considered to be within the normal range. The blood samples of patients confirmed with jaundice **(n=28)**, in EDTA, were collected from the laboratory of Biochemistry, Kasturba Hospital-Manipal immediately after all the laboratory investigations were completed. The relevant

anthropometric data as well as the hematological parameters of the patients were procured and presented in the table S1.

Jaundice Sample No.	*Age (years)*	*Gender (M/F)*	*Total Bilirubin (mg/dL)*	*Direct Bilirubin (mg/dL)*	*Indirect Bilirubin (mg/dL)*	*Hb (g/dL)*	*Hct (%)*
J 1.	61	F	22.2	15.4	6.8	12.2	34.8
J 2.	32	M	11.5	8.7	2.8	7.7	22.2
J 3.	48	M	35.1	26.6	8.5	9.1	26.2
J 4.	46	F	1.6	0.6	1.0	12.9	40.1
J 5.	78	F	35.1	26.6	8.5	9.1	26.2
J 6.	32	M	23.5	20.1	3.4	11.5	35.2
J 7.	49	M	11.0	7.7	3.3	8.6	25.9
J 8.	38	M	24.6	18.3	6.3	11.6	34.5
J 9.	54	M	24.2	18.0	6.2	7.1	21.0
J 10.	41	M	32.3	22.2	10.1	10.5	31.2
J 11.	40	M	13.3	8.7	4.6	7.6	21.9
J 12.	40	M	25.4	17.7	7.7	11.5	33.1
J 13.	60	M	23.1	16.5	6.6	8.3	24.9
J 14.	30	M	19.5	15.1	4.4	9.9	29.2
J 15.	30	M	14.3	11.9	2.4	9.6	28.0
J 16.	40	M	24.3	21.2	3.1	10.6	31.0
J 17.	19	M	16.3	12.1	4.2	8.3	24.3
J 18.	56	M	17.4	15.0	2.4	12.1	35.8
J 19.	29	M	7.4	2.8	4.6	12.0	35.2
J 20.	45	M	37.2	27.7	9.5	10.4	30.2
J 21.	20	F	2.4	1.9	0.5	11.9	35.6
J 22.	48	M	15.1	11.5	3.6	9.5	29.6
J 23.	46	M	33.3	26.6	6.7	9.9	28.8
J 24.	48	M	15.1	11.5	3.6	9.3	27.9
J 25.	44	M	4.5	3.6	0.9	12.7	37.8
J 26.	45	M	26.0	19.2	6.8	8.8	25.0
J 27.	26	M	3.5	0.5	3.0	15.1	44.5
J 28.	70	F	2.1	0.9	1.2	10.7	31.9

Supplementary Table S1: Relevant anthropometric data and hematological parameters of jaundice patients. Patients confirmed with hyperbilirubinemia and above 18 years of age were included in the study. The normal range of total bilirubin, direct bilirubin, indirect bilirubin, Hb counts and Hct are 0.3-12mg/dL (Diazonium method), 0.0-0.4mg/dL (Gendrassik Grof method), 0.3-1.9mg/dL (calculated), 13-17g/dL (photometry) and 40.0%-50.0% (calculated).
